# Supplementary material for: A chromosome-level genome assembly of Zasmidium syzygii isolated from banana leaves
Source: G3 (Bethesda). 2023 Nov 16;14(3):jkad262. doi: 10.1093/g3journal/jkad262 (PMC10917495; doi:10.1093/g3journal/jkad262)
Supplement: jkad262_Supplementary_Data [file jkad262_supplementary_data.docx]

Supplementary Information

# **A chromosome-level genome assembly of *Zasmidium syzygii* isolated from banana leaves**

A.C. van Westerhoven^1,2*^, R. Mehrabi^1,3*^, R. Talebi^3^, M.B.F Steentjes^1^, B. Corcolon^4^, Pablo A. Chong^5^, G.H.J. Kema^1,#,*^, M. F. Seidl^2,#,*^

^1^Laboratory of Phytopathology, Wageningen University & Research, the Netherlands

^2^Theoretical Biology & Bioinformatics, Department of Biology, Utrecht University, the Netherlands

^3^Keygene N.V, P.O. Box 216, 6700 AE, Wageningen, Netherlands

^4^Research, Information, Compliance Department, Tadeco Inc., Panabo, Philippines

^5^ESPOL Polythecnic University, Escuela Superior Politécnica del Litoral, Centro de Investigaciones Biotecnológicas del Ecuador, Laboratorio de Biología Molecular, Guayaquil, Ecuador

*Authors contributed equally

^#^Corresponding authors: Gert H.J. Kema and Michael F. Seidl

**Table S1**. List of primers used in this study.

| Code | Name | Sequence (3’-5’) | Application |
| --- | --- | --- | --- |
| O1 | ACTF1 | TCCAACCGTGAGAAGATGAC | General for fungi |
| O2 | ACTR1 | GCAATGATCTTGACCTTCAT | General for fungi |
| O3 | Pf-actF | CTCATGAAGATCTTGGCTGAG | Specific for *P. fijiensis* |
| O4 | Pm-actF2 | ACGGCCAGGTCATCACT | Specific for *P. musicola* |
| O5 | Pm-actRb | GCGCATGGAAACATGA | Specific for *P. musicola* |
| O6 | Pe-actR | GAGTGCGCATGCGAG | Specific for *P. emusae* |


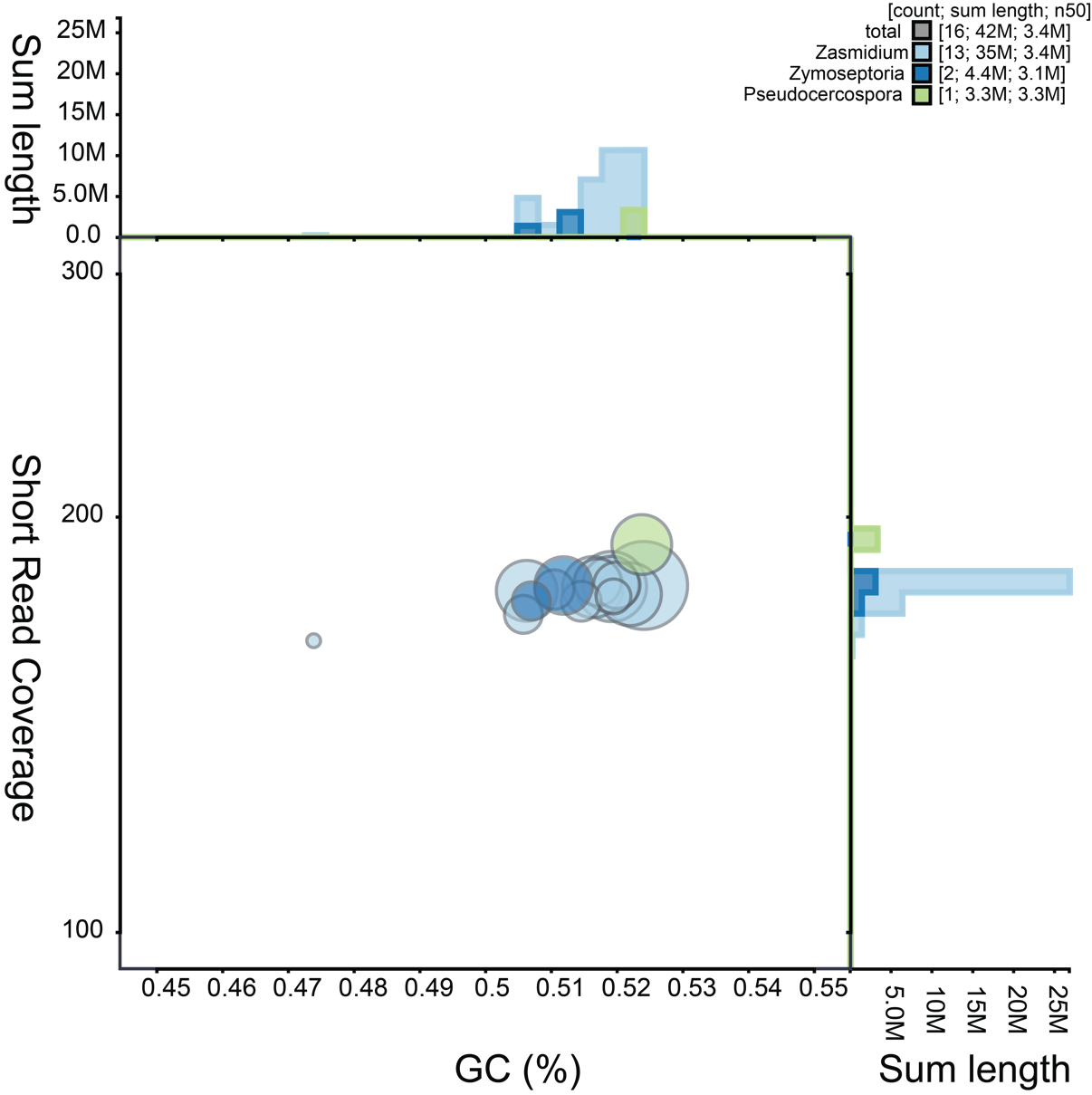


**Figure S1.** Blob plot of base pairs found in the NCBI taxonomy database and the GC content. Fourteen contigs (35 Mb) have the best hits against species from the *Zasmidium* genus, the other 6.4 Mb of the genome have their best hit with other members of Mycosphaerella, Pseudocercospora (3.3 Mb) and Zymoseptoria (3.1 Mb). The GC content of the contigs is around 50% and the contigs are covered by 180 reads on average, suggesting that all contigs belong to a single species.

**
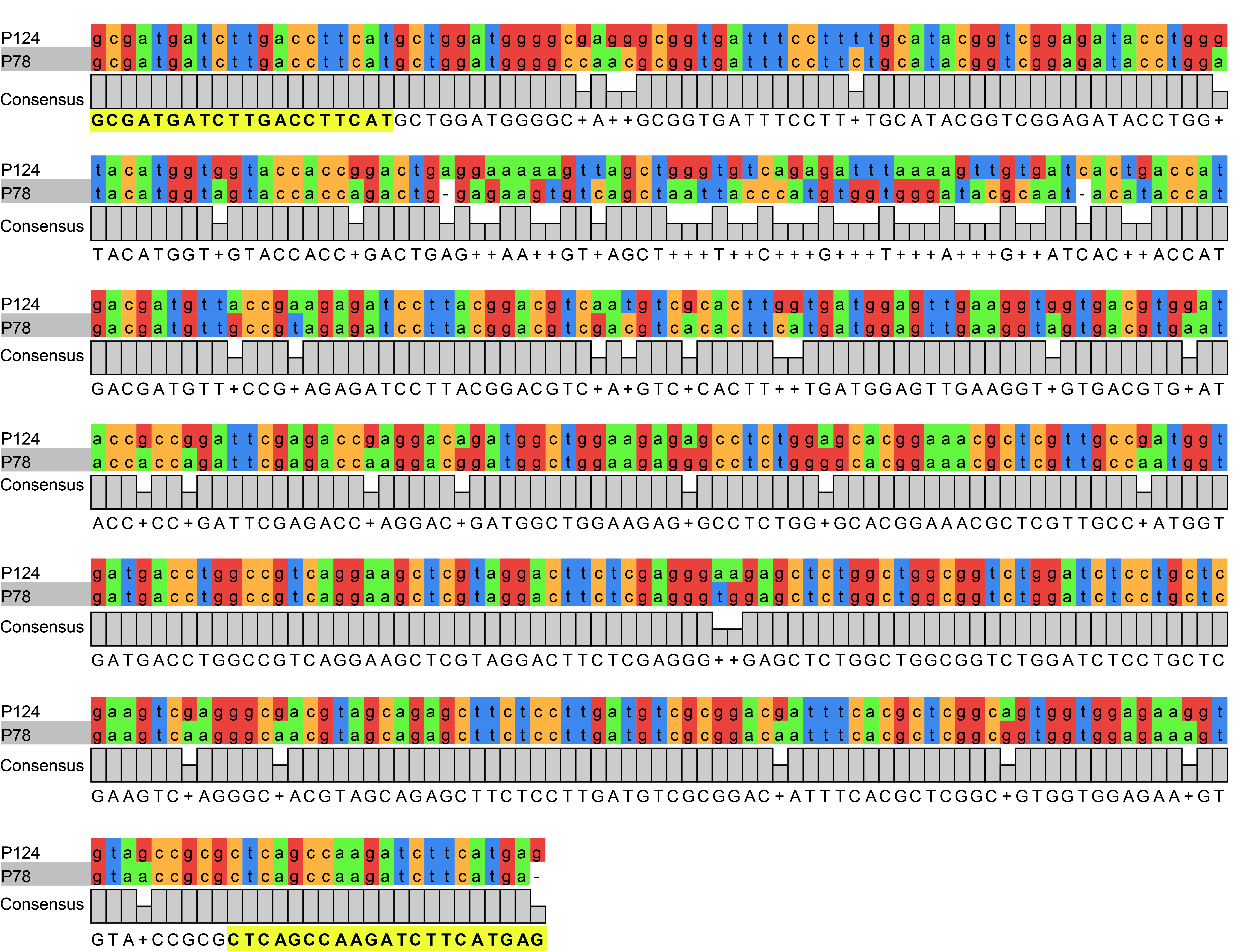
**

**Figure S2.** Nucleotide sequence alignment of the PCR amplicon amplified using the *P. fijiensis*-specific primers. Primer sequence is highlighted in yellow.
